# Comparison between Surgical Outcomes of LASIK with and without Laser Asymmetric Keratectomy to Avoid Conventional Laser Refractive Surgery Adverse Effects

**DOI:** 10.1038/s41598-020-67269-y

**Published:** 2020-06-26

**Authors:** Ji Sang Min, Byung Moo Min

**Affiliations:** 1 Department of Ophthalmology, Yonsei University School of Medicine, Seoul, South Korea; 2 Woori Eye Clinic, Daejon, South Korea

**Keywords:** Health care, Medical research

## Abstract

This study compared one-year postoperative outcomes of laser refractive surgery combined with laser asymmetric keratectomy (LAK) and laser *in situ* keratomileusis (LASIK)for myopia correction in middle-aged patients (aged 40–49 years) with a total corneal thickness deviation (summed across four directions) ≥ 80 microns. The control group (n = 26; 52 eyes) underwent LASIK; the comparison group (n = 26; 52 eyes) underwent combined laser refractive surgery and LAK. Age, spherical equivalence, uncorrected visual acuity (near and far), corneal irregularity on the Orbscan map, sum of corneal thickness deviations in four directions, corneal thickness distribution, distance between the maximum posterior elevation (best-fit sphere; BFS) and visual axis, and postoperative blurring scores were analysed retrospectively between the groups. Both groups had similar preoperative findings. Postoperatively, the sum of corneal thickness deviations in four directions (p = 0.000), distance between maximum posterior elevation (BFS) and visual axis (p = 0.003),blurring score (p = 0.001), and corneal irregularity in the 3.0 and 5.0 mm zones on the Orbscan map (p = 0.033 and p < 0.0001, respectively) were significantly lower in the comparison group (p = 0.000). LAK reduced total corneal thickness deviation, improved corneal symmetry, and reduced blurring scores significantly, one-year postoperatively. LAK could resolve shortcomings of LASIK, producing better surgical outcomes.

## Introduction

Conventional laser refractive surgery, including laser *in situ* keratomileusis (LASIK) and laser epithelial keratomileusis (LASEK), yield good visual corrective effects. However, adverse effects of the surgery, such as reduced visual acuity due to postoperative myopic regression and blurring due to corneal distortion, have also been reported in some patients^1–4^; the postoperative biomechanical changes in the cornea resulting from the interactions between corneal thickness, corneal stiffness, and intraocular pressure laser correction are unavoidable^5–9^. Laser asymmetric keratectomy (LAK)^10^ is a customised biomechanical asymmetric corneal ablation method that has been recently introduced; this method aims to achieve central-symmetric corneal thickness, and the improved corneal shape is maintained long-term without deformation, thus preventing visual abnormalities^10,11^. In this study, we compared the one-year postoperative outcomes of LASIK (conventional method), which uses only ablation to correct refractive power with those of laser correction combined with LAK (a newly introduced method), which is a customised biomechanical method, in middle-aged myopic patients with a corneal thickness deviation (the sum across four directions) ≥ 80 microns.

## Methods

Patients with myopia between the ages of 40 and 49 years who received refractive surgery at the Woori Eye Clinic (Daejon, South Korea) between 2008 and 2017and had total corneal thickness deviation (the sum across four directions) ≥ 80 microns in the Orbscan maps were selected. A retrospective analysis was performed to compare the preoperative and one-year postoperative findings between the control group, which consisted of 26 patients (52 eyes) treated with LASIK for myopia, and the comparison group, which consisted of 26 patients (52 eyes) treated with LAK combined with LASIK or LASEK. The study was conducted in accordance with the 1964 Declaration of Helsinki and its later amendments and was approved by the Korean National Institute for Bioethics Policy (approval number: P01-201911-21-005); the need for informed consent was waived due to the retrospective nature of the study.

**Figure 1:**
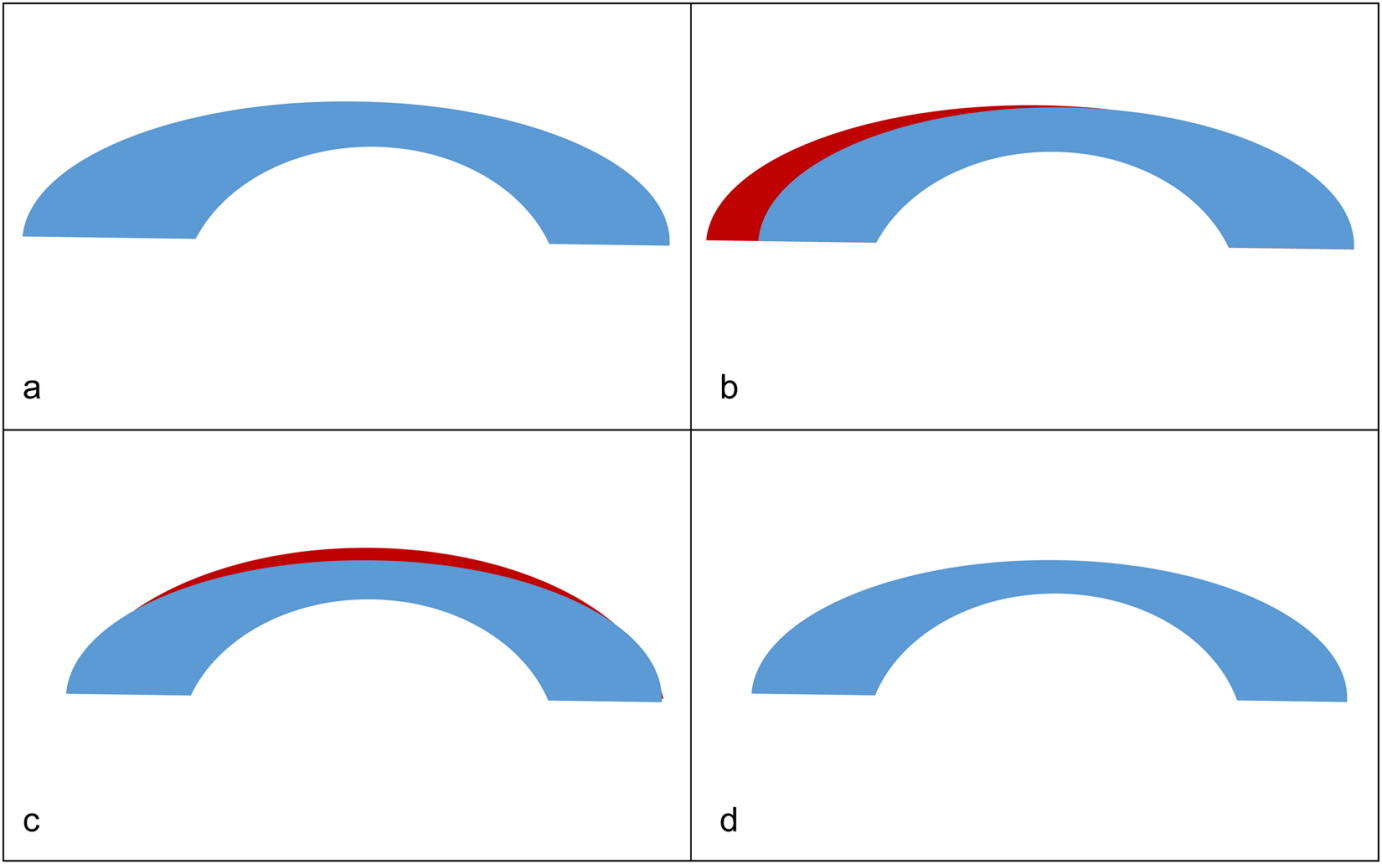
Operative principles of laser asymmetric keratectomy (biomechanical refractive correction). (**a**) Cornea with asymmetric thickness. (**b**) Deviations in the central symmetry of corneal thickness (analysed using Vision-Up software; red area). (**c**) Laser ablation for myopia correction, counterbalancing the myopic corneal curvature caused by ablation of central symmetry deviations, as predicted by the Vision-Up software (red area). (**d**) Cornea showing deviations in the central symmetry of corneal thickness after steps (**b,c**). (Source: ShapeVision Co., Korea).

Laser correction was performed by the same surgeon using the same method in all patients. After inducing local anaesthesia by instillation of 0.50% proparacaine hydrochloride (Alcaine; Alcon NV, Vilvoorde, Belgium) for LASIK, a 9.0–9.5-mm-diameter flap was made using a Moria M2 Keratome (MoriaInc, Antony, France); for LASEK, a 9.0–9.5-mm-diameter patch of corneal epithelia was removed using a brush. For refractive correction, laser ablation was performed in the 6.5-mm optic zone to correct myopia and astigmatism, retaining 2.0 dioptres (D) of myopia; ablation was performed (18 ± 2 microns) in the central 4.5-mm optic zone to create emmetropia within the zone and retain 2 D of myopia outside of the 4.5-mm zone for near vision^12–14^. In terms of laser ablation for LAK, both LAK and laser refractive surgery were performed in patients with refractive abnormalities, while only LAK was performed to create a central symmetry in patients with asymmetric cornea(s) without refractive abnormalities. LAK was performed (as shown in Fig. 1a) for asymmetric corneas with thickness deviations in the central symmetry. Corneal thickness was analysed using the Vision-Up program (WellC Inc., Hwaseong, South Korea), as shown in Fig. 1b. Based on these data, LAK was performed to selectively ablate only the thicker parts of the cornea, creating a central symmetry of corneal thickness.

LAK was performed in two stages of ablation. First, after ascertaining the deviations in central symmetry based on the Vision-Up program, the deviations were eliminated (Fig. 1b). The corneal curvature, which shows myopic shifts due to the corneal ablation procedure, can be predicted by the Vision-Up program. Thus, additional ablation for myopia correction was performed to ensure that the first procedure did not cause a myopic shift (Fig. 1c). This ablation procedure (Fig. 1d) ensured that the resulting corneas were centrally symmetric and of uniform thicknesses.

Thereafter, laser ablation was performed for refractive correction. The target corrected refractive power was −0.75 ± 0.25 D. The laser used for corneal sculpting was a 193-nm ISOBEAM laser (Kera Harvest Inc., Chiayi, Taiwan). A commercially available Argon Fluoride ISOD200 excimer laser device (Kera Harvest Inc., Chiayi, Taiwan) with a wavelength of 193 nm was used for LAK. The laser system has a dual spot size of approximately 0.63 nm^2^, 300 Hz maximum repetition rate, 100–189 mJ/cm^2^ fluence range, less than 1 mJ energy, and a 300-Hz passive type eye tracking function.

A subjective rating^15^ of blurring was recorded using a subjective scale, where 0 = none, 1 = mild, 2 = moderate, and 3 = severe or disturbing, was scored (Table 1), and a retrospective analysis was performed on the pre- and postoperative uncorrected near and far visual acuity, spherical equivalent (SE), blurring severity, corneal irregularity (diopters) in the 3.0 and 5.0 mm zones on the Orbscan map, the sum of deviations in corneal thickness in four directions based on the Orbscan maps, and the distance between the maximum posterior elevation (best-fit-sphere; BFS) and the visual axis. Uncorrected distance visual acuity (UDVA) was measured at a 3 m distance and uncorrected near visual acuity (UNVA) was measured at a 35 cm distance using the Han Chun Suk distance visual acuity chart and the Han Chun Suk near visual acuity chart, respectively. Both UDVA and UNVA were converted to logMAR visual acuity for statistical analysis.

**Table 1:** Blurring scores based on a medical interview.

Score	Extent of blurring
0	No blurring
1	Mild blurring, able to perform daily activities and drive at night
2	Moderate blurring, discomfort in daily activities and driving at night
3	Severe blurring, severe discomfort in daily activities and unable to drive at night

**Figure 2:**
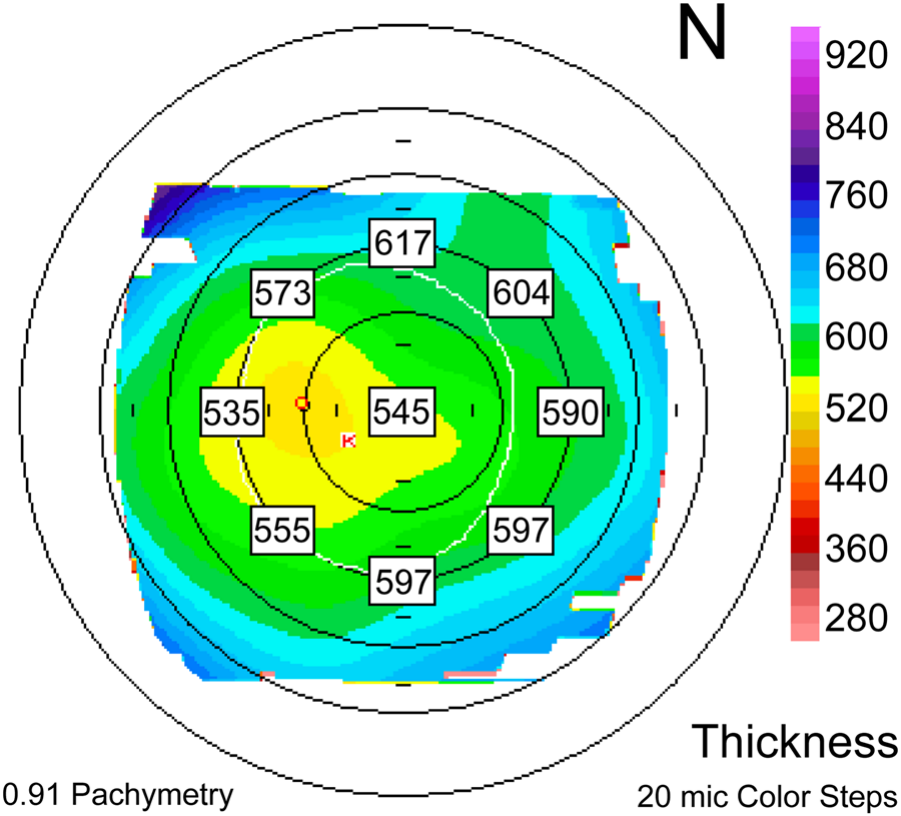
An example for measuring the differences in thickness between symmetrically opposed points (0–180°, 45–225°, 90–270°, and 135–315°). The pachymetric map: 0–180°: 55 microns; 45–225°: 49 microns; 90–270°: 20 microns; 135315°: 24 microns; total: 148 microns. (Orbscan(B&L) Version 3.14).

**Figure 3:**
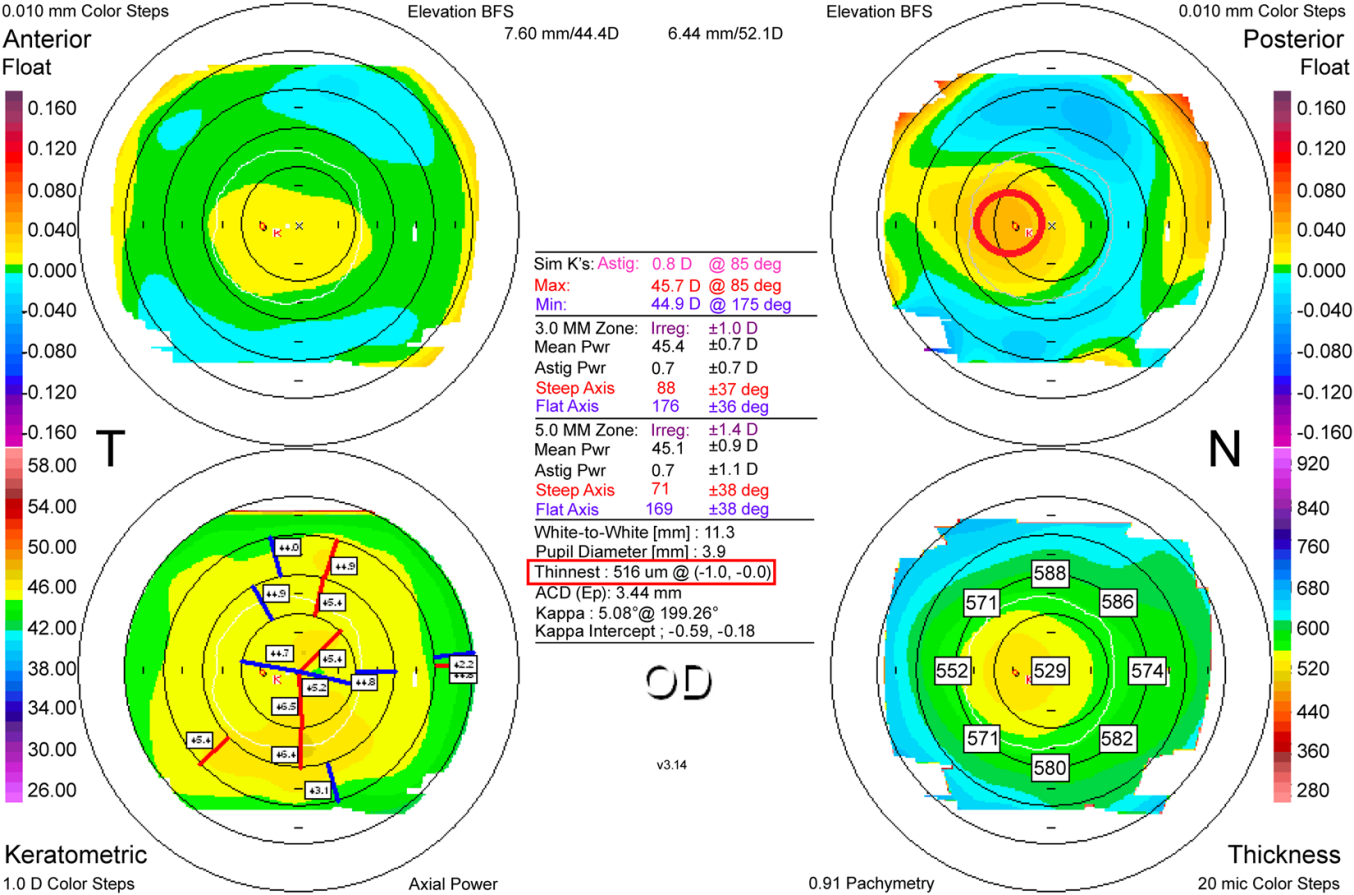
Measuring the distance between the maximum posterior elevation (best-fit-sphere; BFS) and the visual axis. Corneal apex: temporally deviated (right upper red circle). The thinnest point (X, Y) is indicated by the lower red square (Orbscan(B&L) Version 3.14).

Refraction was measured using an auto refractometer/keratometer and calculated as the SE. Pre- and postoperative deviations in corneal thickness were analysed using the following method: first, the thickness was measured using the Orbscan maps at eight locations 2.5 mm from the centre of the cornea (0°, 45°, 90°, 135°, 180°, 225°, 270°, and 315°) and the difference in thickness between symmetrically opposed locations was calculated in four directions (0–180°, 45–225°, 90–270° and 135–315°). The total sum of the differences was then calculated (Fig. 2). The distance between the maximum posterior elevation (BFS) and visual axis was analysed by conversion to the distance between the X and Y coordinates of the thinnest point and the centre of the cornea on the Orbscan map (Fig. 3). In addition, the changes in the distribution of corneal thickness were compared in the two groups between the preoperative and one-year postoperative time points. For statistical analyses, independent samples *t*-tests were performed using the IBM Statistical Package for the Social Sciences program, version 18.0 (IBM Corp., Armonk, NY, USA). P-values <0.05 were considered statistically significant. Data are presented as the mean ± standard deviation unless otherwise stated.

## Results

In terms of preoperative findings (Table 2), the mean age was 45.46 ± 6.88 years in the control group and 43.57 ± 5.30 years in the comparison group (p = 0.250) and the SEs (D) were −3.65 ± 1.68 in the control group and −3.73 ± 1.70 in the comparison group (p = 0.222). For visual acuity (logMAR), the uncorrected far visual acuity was 0.83 ± 0.27 in the control group and 0.78 ± 0.42 in the comparison group (p = 0.315), while the UNVA was 0.48 ± 0.35 in the control group and 0.42 ± 0.27 in the comparison group (p = 0.083); these were all similar between the two groups. The corneal irregularities (diopters) in the 3.0- and 5.0-mm zones on the Orbscan map were 1.44 ± 0.59 and 2.06 ± 2.38 in the control group and1.41 ± 0.44 and 1.60 ± 0.36 in the comparison group (p = 0.779, p = 0.235, respectively (Table 3). The male-to-female ratio was 1:1 in the control group and 6:7 in the comparison group, and the sum of corneal thickness deviations in four directions (microns) was 118.12 ± 34.09 in the control group and 109.13 ± 29.09 in the comparison group (p = 0.358). The distance between the maximum posterior elevation and visual axis was 0.58 ± 0.59 in the control group and 0.62 ± 0.61 in the comparison group, which was not statistically significant (p = 0.379).

**Table 2:** Preoperative characteristics of patients in the control and comparison groups.

Item	Control group (PresbyLASIK)	Comparison group (LAK)	P-value
Age (years)	45.46 ± 6.88	43.57 ± 5.30	0.250
Male-to-female ratio	13:13	12:14	
SE (dioptres)	−3.65 ± 1.68	−3.73 ± 1.70	0.222
UDVA	0.83 ± 0.27	0.78 ± 0.42	0.315
UNVA	0.48 ± 0.35	0.42 ± 0.27	0.083
Sum of corneal thickness deviations in four directions	118.12 ± 34.09	109.13 ± 29.09	0.358
Distance between the maximum posterior elevation and visual axis	0.58 ± 0.59	0.62 ± 0.61	0.379

Data are shown as mean ± standard deviation.

Abbreviations: LAK, laser asymmetric keratectomy; PresbyLASIK, presbyopia laser-assisted *in situ* keratomileus; SE, spherical equivalent; UDVA, uncorrected distance visual acuity; UNVA, uncorrected near visual acuity.

**Table 3:** Comparison of corneal irregularity between the control and comparison groups.

Preoperative corneal irregularity (diopters)			
Item	Control group	Comparison group	p-value
3.0 mm zone	1.44 ± 0.59	1.41 ± 0.44	0.779
5.0 mm zone	2.06 ± 2.38	1.60 ± 0.36	0.235
**Postoperative corneal irregularity (diopters)**			
**Item**	**Control group**	**Comparison group**	**p-value**
3.0 mm zone	2.27 = ±3.01	1.19 ± 0.34	0.033
5.0 mm zone	2.31 ± 0.62	1.40 ± 0.39	<0.0001

**Figure 4:**
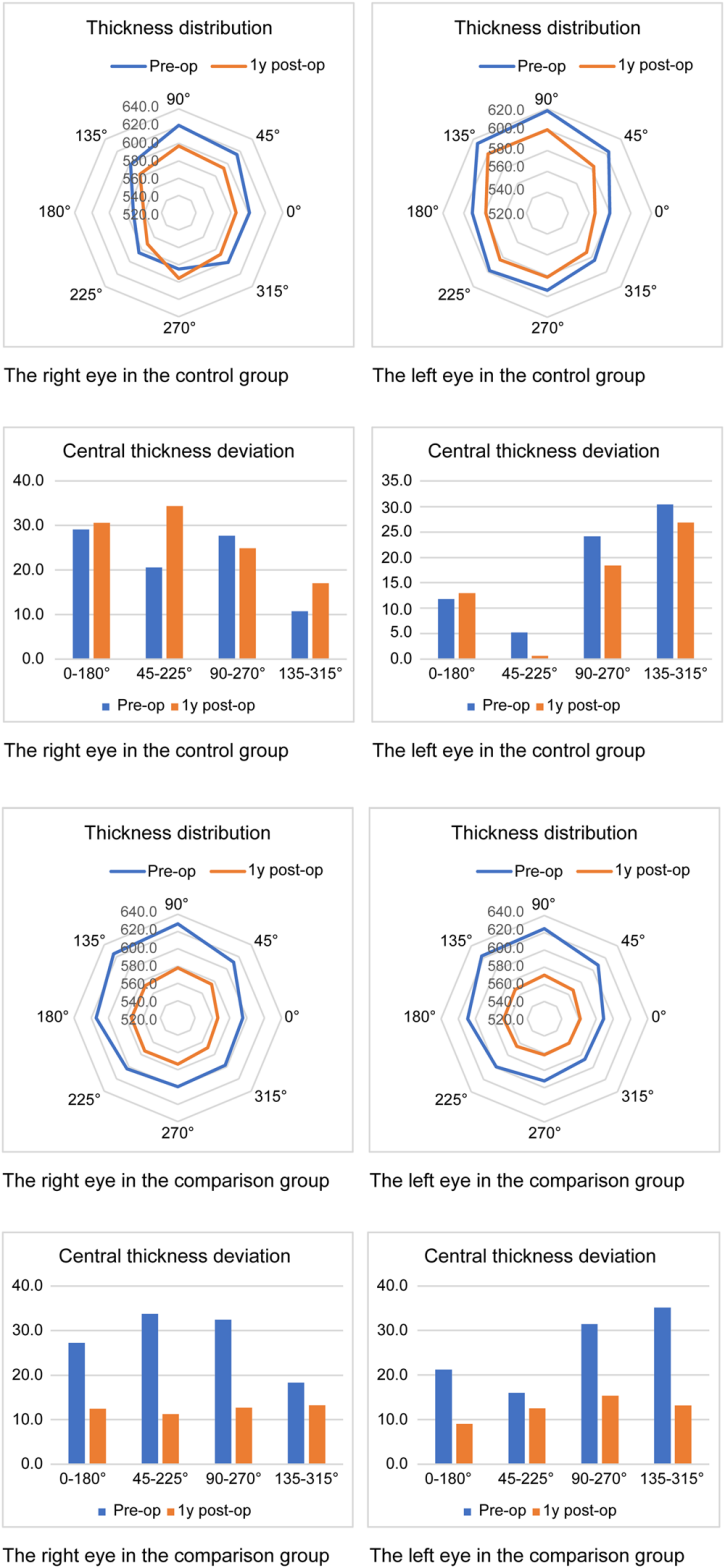
Changes in the distribution of corneal thickness between the preoperative and one-year postoperative time points.

The one-year postoperative findings (Table 4) of the SE(D) was similar between the control (−0.75 ± 0.48) and comparison (−0.63 ± 0.67) groups (p = 0.344). The UNVA (logMAR) was also similar between the control (0.24 ± 0.10) and comparison (0.22 ± 0.13) groups (p = 0.474). The uncorrected far visual acuity (logMAR) was significantly better in the comparison group (0.04 ± 0.07) than in the control group (0.12 ± 0.13) (p = 0.000). The best corrected far vision was 20/20, and near vision was higher than 20/30 for all the patients. The corneal irregularities (diopters) in the 3.0 and 5.0 mm zones on the Orbscan map were 2.27 ± 3.01 and 2.31 ± 0.62 in the control group and 1.19 ± 0.34 and 1.40 ± 0.39 in the comparison group (p = 0.033, p = 0 < 0.0001, respectively) (Table 3). The sum of deviations in the corneal thickness in four directions (microns) also differed significantly between the control (120.04 ± 35.28) and comparison groups (50.02 ± 20.77) (p = 0.000). The distance (mm) between the maximum posterior elevation and visual axis was 0.55 ± 0.53 in the control group and 0.38 ± 0.18 in the comparison group (p = 0.003), while the blurring score was 2.54 ± 0.61 in the control group and 0.33 ± 0.47 in the comparison group (p = 0.001), both being significantly lower in the comparison group than in the control group. The change in the distribution of corneal thickness following surgery was minimal in the control group; however, a large decrease was observed in the comparison group (Fig. 4).

**Table 4:** Summary of 1-year postoperative findings in the control and comparison groups.

Item	Control group (PresbyLASIK)	Comparison group (LAK)	P-value
SE (dioptres)	−0.75 ± 0.48	−0.63 ± 0.67	0.344
UDVA	0.12 ± 0.13	0.04 ± 0.07	0.000
UNVA	0.24 ± 0.10	0.22 ± 0.13	0.474
Sum of corneal thickness deviations in four directions	120.04 ± 35.28	50.02 ± 20.77	0.000
Distance between the maximum posterior elevation and the visual axis	0.55 ± 0.53	0.38 ± 0.18	0.003
Blurring score	2.54 ± 0.61	0.33 ± 0.47	0.001

Data are shown as mean ± standard deviation

Abbreviations: LAK, laser asymmetric keratec.tomy; PresbyLASIK, presbyopia laser-assisted *in situ* keratomileus; SE, spherical equivalent; UDVA, uncorrected distance visual acuity; UNVA, uncorrected near visual acuity.

## Discussion

In this study, both groups preoperatively showed a sum of corneal thickness deviations above 80 microns in four directions on the Orbscan map. However, the control group comprised patients who had complaints of blurry vision, with scores of 2–3 (average: 2.54) after LASIK. While the comparison group comprised patients, who had complaints of blurry vision and had scores of 0–1 (average: 0.33) after LASIK combined with LAK. Blurring scores were rated subjectively; however, corneal irregularities on the Orbscan map in the 3.0 mm and 5.0 mm zones were less in the comparison group when objectively estimated. The two groups were similar in terms of preoperative age, sum of the corneal thickness deviations in four directions, SE, uncorrected near and far visual acuities, and distance between the maximum posterior elevation and visual axis. Both LASEK and LASIK^12^ involve the use of laser to induce symmetric ablation of the corneal parenchyma to achieve the desired refractive correction in the central 8.5 mm optical zone. Therefore, other than the minute changes due to epithelial recovery of the corneal flap and changes in the corneal thickness due to laser ablation, there are no postoperative changes in the factors maintaining the corneal morphology, such as the physicochemical properties of the cornea and the intraocular pressure. Thus, the theoretical changes in the cornea after surgery should be within a predictable range, with the cornea retaining a similar shape to that before surgery, only thinner due to symmetric ablation. However, as reported previously^1–4^, the actual outcomes show adverse effects, such as blurred vision and an altered postoperative corneal shape and curvature. The group that received LAK showed large decreases in the deviations of the central asymmetry in the distance between the maximum posterior elevation and visual axis, and in the blurring scores (p = 0.001). This suggests that LAK is highly effective for achieving corneal point symmetry^10,11^.

We performed LASIK for myopia using an optical method that creates a myopic curvature in the peripheral zone^12^. However, this led to visual abnormalities, such as postoperative blurring, and many patients experienced corneal deformation and reduced visual acuity. Due to the deviations in corneal thickness, the thinner parts of the cornea are expected to undergo biomechanical changes as a result of the intraocular pressure. Recently, a study found that 20%–41% of patients reported adverse effects, such as blurring, dry eyes, and visual aberrations, one year after LASIK^4^. In our study, the one-year postoperative SE was similar between the control and comparison groups (which received LAK). However, compared to the control group, the LASIK group showed myopic regression over time after one postoperative year. However, because the comparison group showed improved corneal symmetry, these patients are expected to show much lower rates of adverse effects due to corneal deformation in the long term. Unlike previously reported wavefront-guided/topography-guided LASIK or LASEK;^16–18^ LAK^10,11^ (which uses corneal asymmetric ablation) has been predicted to reduce corneal thickness deviations and the distance between the maximum posterior elevation and visual axis, thereby improving corneal symmetry and preventing postoperative biomechanical changes in the cornea^19–25^. Thus, LAK is expected to resolve the shortcomings of LASIK and LASEK, producing better surgical outcomes.

In this study, the comparison group showed better corneal thickness deviation and distribution as well as significantly reduced blurring compared to that in the control group. Further, we found that the one-year postoperative uncorrected far visual acuity was better in the comparison group than that in the control group despite similar SEs, which may be due to the effects of blur reduction. For near visual acuity, this effect is assumed to be reduced due to pupil constriction, but further studies will be required. LAK is a useful corrective method for patients who complain of adverse effects such as blurring due to corneal distortion after glaucoma or cataract surgery, as it can reduce the intraocular pressure and corneal stiffness postoperatively^10,11,26,27^. Moreover, it has been reported that LAK, which only cuts the thick parts of the cornea to create central symmetry, can be useful in reducing the effects of intraocular pressure pushing outwards on the thin parts of the cornea in keratoconus, as well as to lessen the asymmetric morphology of the cornea and to reduce the incidence of optical aberrations^28–32^ LAK, which is a customised biomechanical asymmetric corneal ablation method, has only been in use recently; hence, we could only conduct a comparative study with a one-year follow up, and long-term follow up studies are required in the future.

This study has several limitations that should be considered when interpreting the results. First, it was a single centre retrospective study; therefore, there may have been some selection bias. Second, the sample size was relatively small. Finally, only Korean patients were included; thus, the findings may not be generalisable to other ethnic groups.

In conclusion, laser refractive correction combined with LAK resulted in improved corneal thickness deviations and distributions and significantly reduced blurring at one year postoperatively. In future, we anticipate further research on LAK and its treatment outcomes, which will help determine its most useful features. However, we believe that the indications for LAK will include advanced biomechanical customised refractive surgery, treatment of corneal distortions after intraocular surgery (e.g. cataract or glaucoma operations), and treatment of early keratoconus and posterior corneal ectasia.

## Data Availability

The datasets generated during and/or analysed during the current study are not publicly available due to unavailability of ethical consent/approval for transfer of patient data to third parties, institutional mandate/guidelines but are available from the corresponding author on reasonable request.
